# Assessing and Interpreting the Metagenome Heterogeneity With Power Law

**DOI:** 10.3389/fmicb.2020.00648

**Published:** 2020-05-06

**Authors:** Zhanshan (Sam) Ma

**Affiliations:** ^1^Computational Biology and Medical Ecology Lab, State Key Laboratory of Genetic Resources and Evolution, Kunming Institute of Zoology, Chinese Academy of Sciences, Kunming, China; ^2^Center for Excellence in Animal Evolution and Genetics, Chinese Academy of Sciences, Kunming, China

**Keywords:** metagenome ecology, metagenomic gene abundance (MGA) table, Taylor’s power law, power law extensions, metagenome spatial heterogeneity, metagenome functional gene cluster (MFGC), medical ecology of metagenome

## Abstract

There are two major sequencing technologies for investigating the microbiome: the amplicon sequencing that generates the OTU (operational taxonomic unit) tables of marker genes (e.g., bacterial 16S-rRNA), and the metagenomic shotgun sequencing that generates metagenomic gene abundance (MGA) tables. The OTU table is the counterpart of species abundance tables in macrobial ecology of plants and animals, and has been the target of numerous ecological and network analyses in recent gold rush for microbiome research and in great efforts for establishing an inclusive theoretical ecology. Nevertheless, MGA analyses have been largely limited to bioinformatics pipelines and *ad hoc* statistical methods, and systematic approaches to MGAs guided by classic ecological theories are still few. Here, we argue that, the difference between “gene kinds” and “gene species” are nominal, and the metagenome that a microbiota carries is essentially a ‘community’ of metagenomic genes (MGs). Each row of a MGA table represents a metagenome of a microbiota, and the whole MGA table represents a ‘meta-metagenome’ (or an assemblage of metagenomes) of *N* microbiotas (microbiome samples). Consequently, the same ecological/network analyses used in OTU analyses should be equally applicable to MGA tables. Here we choose to analyze the heterogeneity of metagenome by introducing classic Taylor’s power law (TPL) and its recent extensions in community ecology. Heterogeneity is a fundamental property of metagenome, particularly in the context of human microbiomes. Recent studies have shown that the heterogeneity of human metagenomes is far more significant than that of human genomes. Therefore, without deep understanding of the human metagenome heterogeneity, personalized medicine of the human microbiome-associated diseases is hardly feasible. The TPL extensions have been successfully applied to measure the heterogeneity of human microbiome based on amplicon-sequencing reads of marker genes (e.g., 16s-rRNA). In this article, we demonstrate the analysis of the metagenomic heterogeneity of human gut microbiome at whole metagenome scale (with type-I power law extension) and metagenomic gene scale (type-III), as well as the heterogeneity of gene clusters, respectively. We further examine the influences of obesity, IBD and diabetes on the heterogeneity, which is of important ramifications for the diagnosis and treatment of human microbiome-associated diseases.

## Introduction

Understanding the microbiome or “the biome of microbes” usually starts with cataloging the list of OTUs (operational taxonomic units) and tabulating their abundance distribution, leading to the so-termed OTU table. The OTU table has a counterpart in macrobial ecology of plants and animals, known as species abundance distribution (SAD). The recognition of the equivalence between OTU table (or OTU distribution) and SAD has greatly facilitated the infiltration of macrobial ecology theories into microbial ecology. The translation and testing of the ecological theories originated in macrobial ecology with microbiome datasets also lead to the ongoing development of a *unified* or *inclusive* ecology of plants, animals and microbes. Of course, OTU tables, which are usually obtained through amplicon sequencing of marker genes (e.g., 16S-rRNA for bacteria or 18S-rRNA for fungi), are not sufficient for understanding microbiome. For this reason, scientists investigate the metagenome (i.e., the total genomes of all microbes in a microbiome) by using the whole-genome or metagenome shotgun (MGS) sequencing technology. The output from the MGS sequencing technology is the metagenomic gene abundance (MGA) table, which is rather similar to the OTU table, given that both are the abundance of genes (i.e., 16S-rRNA gene *vs*. regular genes). Nevertheless, there is an essential difference between the OTU table and MGA table: the OTU table carries taxonomic information, but MGA table carries genetic or gene information. The former has been a *de fact* standard entity in ecological analyses of the microbiome datasets, and the latter has been mostly used in genetic and evolutionary analyses. In existing metagenomic research, however, few ecological analyses have been performed with MGA data. We argue that the ecological analysis of metagenomic MGA, or “the ecology of metagenome,” is an emerging field where ecological theories should play a critical role.

The similarity between OTU and MGA tables is far from superficial. The familiar OTU table is a matrix of OTU *reads* that capture the species abundance distribution (SAD) of all species in *N* microbial communities (e.g., *N* microbiome samples from *N* individuals, spatial sites or time-points of an individual), with each row corresponding to the SAD of each species, which is simply the frequency distribution (relative abundance) of an OTU across *N* samples. Together, an OTU table represents a meta-community or ecosystem (when meta-factors were added as special columns) in terms of species abundance distribution, including both taxonomic identities and their population abundances in the system. Various ecological analyses (theories and models) such as diversity analysis, power law, diversity-area relationship (DAR), neutral theory and network analyses have been conducted with OTU tables, to reveal important insights on the structure, dynamics and functions of microbiomes (e.g., Costello et al., 2012; Lozupone et al., 2012; Hanson et al., 2012; Human Microbiome Project Consortium [HMP], 2012; Barberaìn et al., 2014; Ma, 2015, 2018, 2019; Ma and Li, 2018, 2019; Li and Ma, 2019; Ma and Ellison, 2019; Ma et al., 2019). These analyses have become a *de facto* standard for 16S-rRNA based (amplicon-sequencing based) microbiome research. However, few such analyses have been applied to MGA tables.

Conceptually, if we conceive the metagenomic genes as “gene species,” then these gene species or genes (we use both the terms interchangeably hereafter) constitute “a community of gene species,” which is essentially the concept of *metagenome*. Each metagenome constitutes one row of a MGA table. In other words, a MGA table consists of multiple (*N*) metagenomes, corresponding to *N* metagenome samples, and a MGA table can be considered as an assemblage or meta-community of metagenomes. Here we coin the term “*assemblage of metagenomes*” (=*metagenome assemblage*) or “*assemblage*” (when no confusion occurs) to represent “metacommunity of metagenomes” or ‘meta-metagenome’ and also to avoid the double prefix of ‘meta-’. Therefore, a MGA table represents an assemblage of metagenomes, consisting of *N* metagenomes, e.g., from *N* individuals (or samples). When meta-factors (such as host physiology) are added to a MGA table, then the MGA table describes an “ecosystem of metagenomes.” With such conceiving, we argue that ecological and network analyses can be harnessed to investigate important problems in metagenome research such as diversity (Ma and Li, 2018), heterogeneity, functional redundancy, mechanisms of diversity maintenance, inter-gene interactions, and dynamics of metagenomes. In a previous study, we successfully demonstrated the application of Hill numbers for measuring metagenome diversity and similarity (Ma and Li, 2018). In this study, we demonstrate the application of Taylor’s power law (Taylor, 1961, 1984; Taylor and Taylor, 1977) and its recent extensions to community ecology (Ma, 2015) to assess and interpret the *heterogeneity* of metagenome assemblage.

According to Li and Reynolds (1995) *heterogeneity* can be defined based on two components: the system property of interests and its complexity or variability. They defined heterogeneity as “the complexity and/or variability of a system property in space and/or time” (Li and Reynolds, 1995). To some extent, considering heterogeneity as the other side of evenness coin or as a proxy of biodiversity is not unreasonable. However, if we look into its usage in population ecology, specifically in the studies on the population spatial distribution of animal or plants, we may quickly recognize one significant difference in community heterogeneity and community diversity. That is, the former is either explicitly or implicitly associated with certain spatial elements, but the latter is not, arguably, beta-diversity is an exception. In addition, heterogeneity is a “group” property in the sense that comparing heterogeneity generally requires at least two entities. As a side note, the heterogeneity in time (states) or temporal heterogeneity is similar to (temporal) stability (Ma, 2015) and is not a topic of this study. In the following, we use the term *heterogeneity* to refer to *spatial heterogeneity* whenever confusion is unlikely.

In the following, we demonstrate the assessment and interpretation of the metagenomic heterogeneity of human gut microbiome at whole metagenome scale with type-I power law extension (PLE) and metagenomic gene scale (type-III PLE), as well as the heterogeneity of functional gene clusters, respectively. Here, the term *spatial* can be applied to different individuals or different microbiome habitats of an individual in the case of human microbiomes, or samples from different habitats in the case of general environmental metagenomes. Furthermore, we also investigate the influence of three common microbiome associated diseases (obesity, diabetes, and IBD) on the metagenomic spatial heterogeneity in human gut systems.

## Concepts and Definitions

**FIGURE 1:**
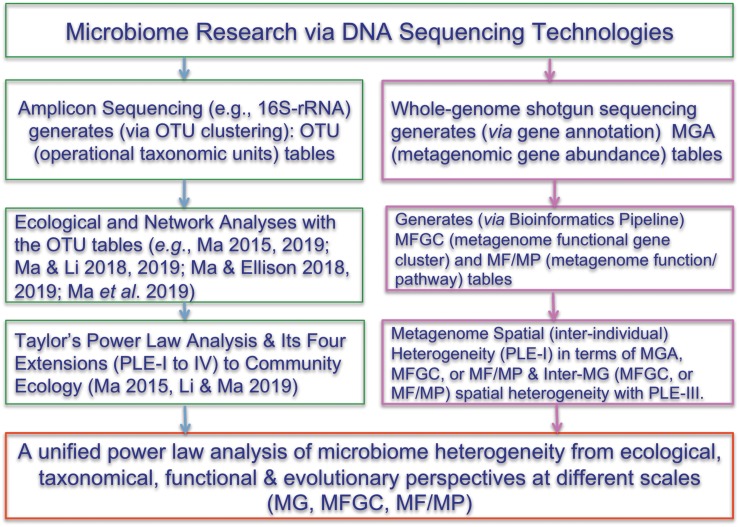
Showing the flowchart of analyzing the microbiome heterogeneity from ecological, taxonomical, functional and evolutionary perspective in terms of various scales [OTU, MG (metagenomic gene), MFGC (metagenome functional gene clusters), MF/MP (metagenomic function/pathway) with the power law extensions (PLEs)]. The right side and framed in red color are newly introduced in the present study. See the Online Supplementary Information (OSI) for the R-Scripts implementing the PLE analysis and randomization tests.

One of the most important findings that the Human Microbiome Project Consortium [HMP] (human microbiome project) has revealed is the enormous inter-subject difference or heterogeneity among individual subjects. However, much of the evidence supporting the notion of personalized microbiome comes from 16S-rRNA datasets. This is because the OTU tables generated from 16S-rRNA sequencing are inherently more submissive to ecological analyses than the MGA tables generated from the whole-genome metagenomic sequencing are. Indeed, compared with the analysis of 16S-rRNA OTU tables, the applications of ecological theories (laws) to the metagenome MGA data analysis have been much fewer. Here, we propose to introduce Taylor’s power law (Taylor, 1961, 1984, 2007; Taylor and Taylor, 1977; Taylor et al., 1983, 1988) and its recent extensions (Ma, 2015; Oh et al., 2016) to the ecological community, for assessing and interpreting the spatial (or inter-subject) heterogeneity within the metagenome assemblage represented by a MGA table. Figure 1 below shows the flowchart of various ecological and bioinformatics analyses involved in the present study.

Taylor’s (1961) power law, describing the scaling relationship between the population mean abundance (*m*) and its variance (*V*) over space (i.e., *V* = *a*^*mb*^), is one of few well recognized ecological laws in population ecology, and it offers a powerful mathematical tool to measure the spatial *aggregation* (*heterogeneity*). Its power law scaling parameter (*b*) often embodies rich ecological and evolutionary insights about specie abundance and distribution over space or time across different environments (Taylor, 1961, 1984, 2007; Taylor and Taylor, 1977; Taylor et al., 1983, 1988). Since its discovery more than a half century ago (Taylor, 1961), Taylor’s power law has been the target of numerous field tests and theoretical analyses, especially in macrobial ecology of plants and animals. In particular, a resurgence of theoretical investigation and extensions to even wider applications in many fields of science and technology, particularly inter-disciplinary studies, has been ongoing in the last few years (e.g., Reuman et al., 2009, 2014, 2017; Cohen et al., 2012, 2013; Ma, 2012, 2015; Stumpf and Porter, 2012; Wearn et al., 2013; Cohen, 2014; Zhang et al., 2014; Cohen and Xu, 2015; Cohen and Saitoh, 2016; Oh et al., 2016; Tippett and Cohen, 2016; Quist et al., 2017). In a previous study (Ma, 2015), we extended the original Taylor’s (1961) power law from population to community level and tested four power law extensions (PLEs) with the 16s-rRNA amplicon-sequencing datasets of the microbial communities from the human microbiome project (Human Microbiome Project Consortium [HMP]). Among the four PLEs introduced by Ma (2015), Type-I and Type-III PLEs can quantify the community (level) spatial heterogeneity and mixed-species (level) spatial heterogeneity, respectively. Type-II and Type-IV were proposed to assess the community temporal stability and mixed-species temporal stability, respectively, but this study does not implicate them since both Type-II and IV require time-series data, for which we did not get sufficiently large datasets, but they should still be applicable for measuring the metagenome stability.

### PLE-I (Type-I Power Law Extension) for Measuring Metagenome Spatial Heterogeneity

Similar to the PLE-I for measuring community spatial heterogeneity (Ma, 2015), we propose to use the following mean-variance power function for measuring the *metagenome spatial heterogeneity* of a metagenome assemblage (or meta-metagenome, as explained previously):

(1) $$V_{s}=a\,m_{s}^{b}\;s=1,2,\ldots ,S$$

where *m*_*s*_ is the mean of gene abundances of all genes (species) (*G*) in the metagenome of an individual subject (*s* = 1, 2, …*S*), *V*_*s*_ is the corresponding variance, *S* is the number of subjects, and *G* is the number of genes contained in the metagenomes of the *S* subjects. Note *m*_*s*_ is the mean gene abundance *per* gene species, not per subject, which is different from the case in PLE-III (type-III power law extension) introduced below. In addition, the fitting of Eqn. (1) is performed with *S* data points, i.e., across *S* individual subjects (or *S* metagenomes), rather than across genes, as in the case of PLE-III below.

The parameter *b* describes the *fractional scaling* of *V*-*m* relationship statistically, or the *metagenome spatial heterogeneity* biologically. When *b* = 1, the heterogeneity is random, which means that the heterogeneity—the inter-subject difference in their gene abundance distribution—is essentially random, statistically follows Poisson distribution. When *b* > 1, the inter-subject heterogeneity is non-random and follows highly skewed long-tail distribution (such as the power law distribution). When *b* < 1, the inter-subject heterogeneity in their metagenome is fixed, or follows the uniform statistical distribution. From field studies in ecology, the cases when *b* = 1 or *b* < 1 are extremely rare in real world and usually only exist theoretically (Taylor, 1961, 1984). We term a metagenome assemblage (i.e., an assemblage of metagenomes) with *b* = 1 random metagenome (strictly speaking, metagenome assemblage), *b* < 1, homogenous metagenome, and *b* > 1, heterogeneous metagenome.

Parameter *a* in Eqn. (1) is meanwhile related to sampling related factors such as sampling unit or sequencing platforms, but is little influenced by biological interactions. Hence, we generally do not attempt to draw biological interpretations from parameter *a* due to the strong influence from sampling. It is noted that parameter *a* also has the same interpretation in PLE-III below.

We further define *critical diversity of metagenome heterogeneity* (CDMH) or *m*_0_ as:

(2) $$m_{0}=\exp\left[\ln\left(a\right)/\left(1-b\right)\right]\;\left(b\neq 1\right)$$

where *a* and *b* are PLE-I parameters from eqn. (1). The CDMH or *m*_0_ is the *mean gene abundance* level (per gene species) at which metagenome spatial heterogeneity is random, and across which the heterogeneity transits to either heterogeneous (when *m* > *m*_0_) or regular (uniform or fixed) (when m < *m*_0_). Since the *mean gene abundance*, although termed abundance, is essentially a measure of *gene diversity* (i.e., the mean abundance of various gene species in a metagenome), we used the term *critical diversity of metagenome heterogeneity*, rather than using the term “*critical abundance.*” The latter is indeed used in the next section for PLE-III, which is the average of single gene abundances from various individuals and consequently the term *abundance* is more appropriate.

### PLE-III (Type-III Power Law Extension) for Measuring Gene-Level Spatial Heterogeneity

Similar to the PLE-III for measuring mixed-species spatial heterogeneity in Ma (2015), we propose to use the following mean-variance power function for measuring the *gene-level* (*inter-gene*, or *mixed-gene*) *spatial heterogeneity*:

(3) $$V_{g}=a\,m_{g}^{b}\;g=1,2,\ldots ,G$$

where *m*_*g*_ is the mean abundance of *g*-*th* gene, averaged across *S* subjects (*g* = 1, 2, …*G*), *V*_*g*_ is the corresponding variance, and *G* is the number of gene kinds (gene species), and *S* is the number of individual subjects sampled. Note *m*_*g*_ is the mean gene abundance of the *g*-th gene species *per* subject (not *per* gene), which is opposite from the case in the previously introduced PLE-I for measuring metagenome spatial heterogeneity. In addition, the fitting of Eqn. (3) is performed with *G* data points, i.e., across all G gene species, rather than across *S* subjects, as in the case of the previous PLE-I.

Note that the notion of “mixed-gene” is similar to the concept of mixed-species population in the original Taylor’s power law (Taylor and Woiwod, 1982; Taylor, 1984). It refers to a virtually “averaged assemblage” of genes, in which the identities or kinds of different genes were ignored. The *m*-*V* pairs are regressed (see below, through log-linear transformation into linear regression) across multiple gene species (millions in the case of this study) in a *mixture* manner. Given that the notion of gene *species* is not widely used in metagenomic research, we suggest using the term *gene-level* or *inter-gene* heterogeneity, rather than *mixed-gene* heterogeneity in the context of PLE-III.

When *b* = 1, the heterogeneity among metagenomic genes in terms of their gene abundance distributions should be random, i.e., all genes in the metagenome are equivalent to each other in terms of their abundance distribution, similar to the neutrality assumption in the neutral theory of biodiversity. When *b* < 1, the heterogeneity or difference among genes, if any, should be fixed, or follow a uniform distribution statistically. Both the cases of *b* = 1 or *b* < 1 should be extremely rare in real world, and are mostly theoretical possibilities. In practice, *b* > 1 should be the norm rather than the exception for metagenome heterogeneity at the gene-level. When *b* > 1, we say the metagenome is heterogeneous or aggregated in terms of its *gene-level* or *inter-gene* heterogeneity.

We further define critical abundance of gene-level heterogeneity (CAGH) or *m*_0_ as:

(4) $$m_{0}=\exp\left[\ln\left(a\right)/\left(1-b\right)\right]\;\left(b\neq 1\right)$$

where *a* and *b* are PLE-III parameters from eqn. (3). The CAGH or *m*_0_ is the mean gene abundance level (per individual or sample) at which gene-level spatial heterogeneity is random, and across which the heterogeneity transits to either heterogeneous (when *m* > *m*_0_) or regular (uniform or fixed) (when *m* < *m*_0_).

### Statistical Fitting of PLE-I or PLE-III

To fit the power law, including PLE-I and PLE-III, the most commonly used approach is to transform the power law model [eqn. (1) or (3)] into the following linear function:

(5) ln⁡(V)=ln⁡(a)+b⁢ln⁡(m)

where all the variables (*m, V*) and parameters (*a, b*) have the exactly same interpretations as those in eqn. (1) or eqn. (3). Standard linear regression procedure can be applied to fit the model. In fact, there is an advantage for adopting the simple linear transformed regression approach, which is related to an important property of power law, scale-invariance. This property makes parameter *a* less relevant for determining the most important parameter of power law, i.e., the scaling parameter *b* (Ma, 2015). It is for this reason that we choose the simple linear regression approach for fitting all the power law models. This allows us to focus on the scaling parameter (*b*) for assessing and interpreting the metagenome heterogeneity revealed by the metagenomic sequencing data.

As a side note, we may define *metagenome temporal stability* with Type-II PLE or *gene-level temporal stability* with Type-IV PLE, similar to Ma (2015) for community temporal stability or mixed-species temporal stability, but their demonstrations require time-series MGA data obtained with metagenomic (whole-genome or shotgun) sequencing technologies. We failed to find sufficiently long time-series MGA data to demonstrate the PLE-II or PLE-IV models and won’t further discuss the temporal versions of the PLE in this study (Nielsen et al., 2014).

### Bioinformatics Analysis of Metagenomic Sequencing Data

To fit the power law model, one has to first compute MGA tables from metagenomic sequencing raw reads (also known as shotgun or whole-genome sequencing) by using standard bioinformatics software pipelines (e.g., Li and Godzik, 2006; Qin et al., 2010, 2012; Zhu et al., 2010; Chatelier et al., 2013; Li et al., 2014; Xiao et al., 2015, 2016; Wang and Jia, 2016; Sczyrba et al., 2017; Ma and Li, 2018).

Millions of contigs are obtained through the metagenome assembly step. Those millions of contigs are fed into *gene prediction* software and the latter generate a list of non-redundant genes based on the criteria set by ORFs (open reading frames). We term those non-redundant genes as *metagenomic genes* (MGs) or simple genes. MG embodies single-gene-level genetic information, and its number in a typical metagenome sample is in the magnitude of millions (Ma and Li, 2018). The previously defined MGA table is actually the table of MGs.

Directly characterizing or summarizing information from the millions of MGs or MGA tables can be rather challenging. An alternative research strategy is to first group those millions of genes (MGs) into functional gene clusters, and then investigate the properties of the *functional gene clusters*. There are mature bioinformatics algorithms and software pipelines to cluster the millions of MGs into hundreds of MFGCs (metagenome functional gene clusters), and the magnitude of MFGC numbers (hundreds) is much small that that of the MGs (millions) (Ma and Li, 2018). Obviously, the huge reduction in the magnitudes from MGs (millions) to MFGCs (hundreds) should make our measuring metagenomic spatial heterogeneity simpler.

## Demonstration and Discussion

### The Datasets of Metagenomes

We collected three gut metagenome datasets from public domain including, 264 stool samples from overweight and lean individuals (Qin et al., 2010; Chatelier et al., 2013), 145 stool samples from type-2 diabetes and healthy controls (Qin et al., 2012), and 219 stool samples from IBD patients and healthy controls. A total of 628 metagenome samples with their metagenomic gene (MG) catalog and the gene abundance (MGA) tables for each dataset were computed with standard metagenomic analysis pipelines (e.g., Li and Godzik, 2006; Qin et al., 2010, 2012; Zhu et al., 2010; Chatelier et al., 2013; Li et al., 2014; Xiao et al., 2015, 2016; Wang and Jia, 2016; Sczyrba et al., 2017). Furthermore, we defined metagenome functional gene clusters (MFGC) based on Ma and Li (2018) and obtained their abundance tables. Supplementary Table S1 showed more detailed information about the three datasets we use in this paper for demonstrating the application of the power law.

Specifically, after whole-genome (shotgun) sequencing of a metagenome sample, sequencing reads from the fecal samples were processed for quality control, removal of human sequences, assembling, assembly revision and gene prediction by using MOCAT pipeline (Kultima et al., 2012). This pipeline consists of a series of software packages, which can process metagenomes in a standardized and automated manner while improving the quality of assembly and gene prediction at run time. In the pipeline, FASTX Toolkit^1^ was used for quality control; SOAPaligner2 (Li et al., 2009) for identifying human sequences; SOAPdenovo v1.06 (Li et al., 2010) for assembling; MetaGeneMark (Zhu et al., 2010) for gene prediction; CD-HIT (Li and Godzik, 2006) for clustering genes in each cohort.

The details of the data/software/parameters used to compute the MGA tables can be found in the online method of Li et al. (2014). In fact, the MGA tables are available online at: http://meta.genomics.cn/meta/dataTools. Li et al. (2014) annotated the metagenomic genes according to the “Kyoto Encyclopedia of Genes and Genomes” (KEGG) and the “evolutionary genealogy of genes non-supervised orthologous groups” (eggNOG) databases. They further identified a total of 6,980 KEGG orthologous groups (KOs) and 36,489 eggNOG orthologous groups, accounting for 51.6 and 69.3% of the total sequencing reads.

In the online Supplementary Information (OSI), the R-Scripts for implementing the power law analysis and randomization tests for determining the differences in the PLE parameters are provided.

### *Metagenome Spatial Heterogeneity* in Terms of the MGA (Metagenomic Gene Abundance) Spatial (Inter-Subject) Distribution Measured With PLE-I

We first fitted PLE-I (type-I power law extension) with metagenomic gene abundance (MGA) datasets directly in order to measure the metagenome spatial (inter-subject) heterogeneity for each treatment (group) of the three datasets, and the results were listed in Table 1, from which we summarize the following findings:

**TABLE 1:** The parameters of PLE-I (type-I power law extension) for *metagenome spatial heterogeneity*, in terms of the MGA (metagenomic gene abundance).

Power Law Extension (PLE)	Case study	Treatment	*b*	SE(*b*)	ln(*a*)	SE[ln(*a*)]	*m*_0_	*R*	*p*-value	*N*
Type-I PLE for Metagenome Spatial Heterogeneity with MGA	Obesity	Lean	2.012	0.113	3.740	0.337	0.025	0.878	<0.001	95
		Overweight	3.447	0.158	–1.204	0.532	1.636	0.914	<0.001	96
	Type-II Diabetes	Healthy	3.232	0.210	–0.529	0.650	1.267	0.876	<0.001	74
		Disease	1.846	0.143	3.982	0.447	0.009	0.840	<0.001	71
	IBD	Healthy	1.385	0.079	5.365	0.266	0.000	0.903	<0.001	71
		Disease	2.248	0.227	2.754	0.761	0.110	0.766	<0.001	71

**FIGURE 2:**
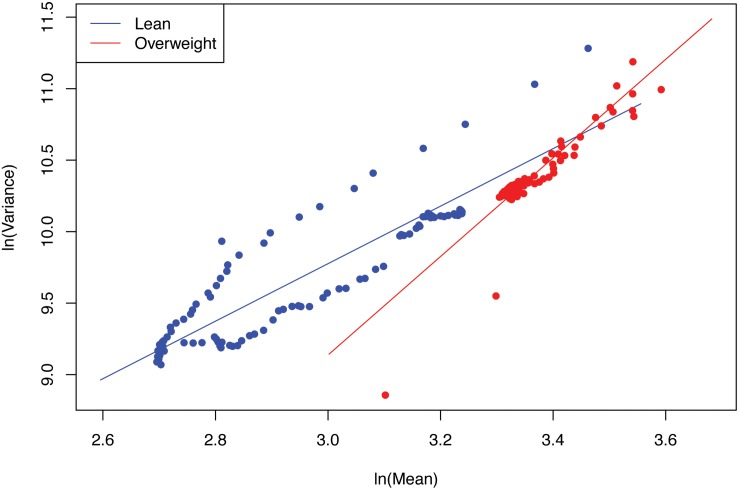
The PLE-I (type-I power law extension) models fitted for the obesity case study.

(i) PLE-I fitted to all three datasets extremely well with *p*-value < 0.0001. This indicates the ubiquitous applicability of the PLE for assessing the metagenome spatial (i.e., inter-subject) heterogeneity of either MGA (this section) or MFGC (the next section).
(ii) The scaling parameter (*b*) of PLE-I for the most treatments is between *2* and *4* except for the two treatments (diseased treatment in the diabetes study, and the healthy treatment in the IBD study), and the parameter (*b*) varied significantly between the treatments with a range of [*1.385, 3.447*].
(iii) The values of the scaling parameter (*b*) for the healthy samples (group) and diseased samples (group) were significantly different (*p*-value < 0.05), in all three case studies (obesity, diabetes and IBD). Therefore, we conclude that PLE-I can be harnessed to measure the *metagenome spatial heterogeneity* in terms of gene abundance distribution. Furthermore, it has a potential being a discriminant metric for distinguishing between the healthy and diseased metagenome samples, as revealed in Table 2 (*p*-value < 0.05), in which randomization test (Collingridge, 2013) with 1000 times of re-sampling was utilized to test the difference in the *b*-value between the healthy and diseased treatments. Figure 2 shows the fitted power law models for the obesity case study, i.e., one straight line for the lean group and another for the overweight group.

**TABLE 2:** The *p*-value of the randomization test for the difference between the healthy and diseased treatments in their metagenome spatial heterogeneities parameters of PLE-I.

Power Law Extension (PLE)	Case Study	Treatments	*b*	ln(*a*)	*m*_0_
Type-I PLE for Metagenome Spatial Heterogeneity with MGA	Obesity	Lean vs. Overweight	<0.001	<0.001	<0.001
	Type-2 diabetes	Healthy vs. Disease	0.044	0.038	0.044
	IBD	Healthy vs. Disease	0.021	0.043	0.015

The *metagenome spatial heterogeneity* is the counterpart of *community spatial heterogeneity* in community ecology, and it measures the spatial heterogeneity of metagenomes of individual subjects or inter-subject metagenome heterogeneity in a population (or cohort), similar to measuring the heterogeneity among spatially explicit local communities in community ecology (Ma, 2015). With our newly coined term of metagenome assemblage, parameter *b* measures the heterogeneity of metagenome assemblage represented by a MGA table. The higher *b*-value of PLE-I represents greater heterogeneity (unevenness or diversity) among individuals in their metagenomes in terms of their gene abundance distributions. When *b* = 1, it implies that the differences among individuals are random. When *b* < 1, it implies that the differences among individuals follow *uniform* distribution statistically (i.e., a fixed difference).

### *Gene-Level (or Inter-Gene) Spatial Heterogeneity* in Terms of the MGA (Metagenomic Gene Abundance) Distribution Measured With PLE-III

We also fitted the PLE-III (type-III power law extension) with metagenomic gene abundance (MGA) datasets directly in order to measure the gene-level or mixed-gene spatial heterogeneity for each of the 3 datasets, and the results were listed in Table 3 below. It was shows that:

**TABLE 3:** The parameters of PLE-III (type-III power law extension) for measuring gene-level (inter-gene) spatial aggregation, in terms of the metagenomic gene abundance (MGA).

Power Law Extension (PLE)	Case study	Treatment	*b*	SE(*b*)	ln(*a*)	SE[ln(*a*)]	*m*_0_	*R*	*p*-value	*N*
Type-III PLE for Gene-Level Spatial Heterogeneity with MGA	Obesity	Lean	2.371	0.000	−0.732	0.001	1.706	0.961	<0.001	5407291
		Overweight	2.363	0.000	−0.744	0.001	1.726	0.961	<0.001	5134721
	Type-II Diabetes	Healthy	2.340	0.000	−0.842	0.001	1.875	0.954	<0.001	4573927
		Disease	2.338	0.000	−0.791	0.001	1.806	0.949	<0.001	4432814
	IBD	Healthy	2.466	0.000	−1.000	0.001	1.978	0.961	<0.001	2898618
		Disease	2.351	0.000	−0.791	0.001	1.796	0.957	<0.001	4462890

**FIGURE 3:**
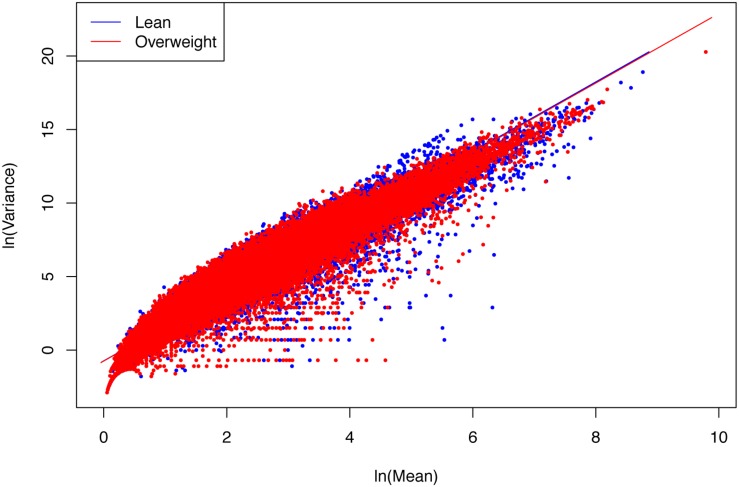
The PLE-III (type-III power law extension) models fitted to the obesity study datasets: more than 10 million points (5407291 lean group + 5134721 overweight) were used to fit the PLE-III models, but here we only randomly selected 100,000 points (50,000 from each treatment) to draw the graphs (so as to accommodate the file size of the figure).

(i) The PLE-III fitted to all 3 datasets extremely significant with *p*-value < 0.0001, and the standard errors of the model parameters were close to zero. The linear correlation coefficients were between 0.949 and 0.961. All the criteria indicate that the goodness-of-fitting to PLE-III was extremely well given millions of data points were fitted.
(ii) The parameter *b* of PLE-III for all the treatments fall in a rather narrow range of [2.340, 2.466]. Therefore, we conclude that PLE-III can be harnessed to measure the gene-level spatial heterogeneity in terms of the gene abundance distribution, but its application for discriminating the healthy and diseased samples is of limited value given its insensitivity to host factors such as diseases. Figure 3 shows the fitted PLE-III with the dataset from the obesity study.

Taylor’s power law has been tested with hundreds, if not thousands, of field studies and many theoretical examinations (Taylor, 1961, 1981, 1984, 2007; Taylor et al., 1983, 1988; Ma, 1991, 2012, 2013, 2015, 2018; Reuman et al., 2009, 2014, 2017; Stumpf and Porter, 2012; Cohen and Xu, 2015; Tippett and Cohen, 2016; Quist et al., 2017). However, to the best of our knowledge, the tests exhibited in Table 3 should be the cases that have used the biggest numbers of data points (the column *N* in Table 3) to fit the power law model, since it was first discovered more than a half century ago. For example, in the case of obesity study, for each of the two treatments (lean *vs*. overweight), more than five million genes were used to fit PLE-III model. This shows the exceptional robustness of the power law model.

The PLE-III for measuring the gene-level or mixed-gene spatial heterogeneity is the counterpart of mixed-species spatial aggregation in community ecology (Ma, 2015). The term *aggregation* is often used in population ecology, and it is the counterpart of heterogeneity in community ecology. As explained previously, the term *mixed-gene* setting assumes that we ignore the identities of individual genes, and what is measured is the aggregation (unevenness or heterogeneity) of an *average gene* species. We suggest using the term “*gene-level spatial heterogeneity”* for what is measured with the PLE-III in metagenomic research.

### Metagenome Spatial Heterogeneity in Terms of the MFGC (Metagenome Functional Gene Cluster) Distribution Measured With PLE-I

According to Ma and Li (2018), the term MFGC (metegenome functional gene cluster) refers to cluster of functionally similar or same genes, generated from functional annotation or gene annotation through online mapping to functional databases such as KEGG (for metabolic pathways) and eggNOG (for protein functions). Hence, MFGC is purely functionality-based and is mostly cross-species. One of its unique advantages is that it embodies the functional redundancy in microbiome very well. The difference between Type-I MFGC (MFGC-I) and Type-II MFGC (MFGC-II) lies in their differences in handling the genes within each cluster. In MFGC-I, only the number of gene species (kinds) is counted but the abundance of individual gene is ignored. In MGGC-II, both the number of gene species (kinds) and the abundance of each gene matter in the analysis. In other words, with MFGC-I, we only care the *number* of gene *species* (kinds), and with MFGC-II we care both the number of *gene species* (*kinds*) and the abundance of each gene within each cluster. This treatment is very similar to a common practice in community ecology, where a simplified measure for biodiversity is to only count the number of species (also known as *species richness*), and a more comprehensive measure of biodiversity uses more sophisticated entropy such as Shannon entropy, which consider both species richness and abundances.

In the previous section, we conducted power law analysis in terms of the metagenomic gene (MG) or metagenomic gene abundance (MGA) distribution. In this section, our analysis is performed in terms of the metagenome functional gene cluster (MFGC). That is, using the *MFGC abundance tables* (similar to MGA or OTU tables, except that the entity is the MFGC) to fit the PLE models.

The results of fitting the PLE-I with MFGC tables were listed in Table 4, from which we can observe the following findings:

**TABLE 4:** The parameters of PLE-I (type-I power law extension) for metagenome spatial heterogeneity, in terms of the MFGC (metagenome functional gene cluster) distribution.

Type of MFGC and database used	Microbiome	Treatment	*b*	*SE*(*b*)	ln(*a*)	*SE*[ln(*a*)]	*R*	*p*-value	*N*	*m*_0_
Type-I MFGC (eggNOG)	Obesity	Lean	2.119	0.020	3.187	0.157	0.996	<0.0001	95	0.058
		Overweight	2.028	0.017	3.895	0.135	0.997	<0.0001	96	0.023
	Type 2 diabetes	Healthy	2.058	0.025	3.501	0.180	0.995	<0.0001	74	0.037
		Disease	2.057	0.018	3.480	0.126	0.997	<0.0001	71	0.037
	IBD	Healthy	2.053	0.017	3.690	0.136	0.998	<0.0001	71	0.030
		Disease	2.138	0.021	3.014	0.159	0.997	<0.0001	71	0.071
MFGC Type-I (KEGG)	Obesity	Lean	2.091	0.015	3.505	0.123	0.998	<0.0001	95	0.040
		Overweight	2.027	0.014	4.008	0.109	0.998	<0.0001	96	0.020
	Type-II diabetes	Healthy	2.035	0.020	3.772	0.150	0.997	<0.0001	74	0.026
		Disease	2.036	0.014	3.794	0.106	0.998	<0.0001	71	0.026
	IBD	Healthy	2.042	0.013	3.888	0.104	0.999	<0.0001	71	0.024
		Disease	2.111	0.016	3.292	0.130	0.998	<0.0001	71	0.052
MFGC Type-II (eggNOG)	Obesity	Lean	1.884	0.021	4.912	0.223	0.995	<0.0001	95	0.004
		Overweight	1.859	0.019	5.212	0.208	0.995	<0.0001	96	0.002
	Type-II diabetes	Healthy	1.783	0.059	5.771	0.614	0.962	<0.0001	74	0.001
		Disease	1.715	0.091	6.461	0.937	0.915	<0.0001	71	0.000
	IBD	Healthy	1.967	0.024	3.992	0.260	0.995	<0.0001	71	0.016
		Disease	1.937	0.021	4.295	0.232	0.996	<0.0001	71	0.010
MFGC Type-II (KEGG)	Obesity	Lean	1.915	0.017	4.834	0.197	0.996	<0.0001	95	0.005
		Overweight	1.889	0.016	5.136	0.178	0.997	<0.0001	96	0.003
	Type-II diabetes	Healthy	1.830	0.054	5.572	0.577	0.970	<0.0001	74	0.001
		Disease	1.806	0.078	5.856	0.831	0.942	<0.0001	71	0.001
	IBD	Healthy	1.988	0.021	3.997	0.234	0.996	<0.0001	71	0.017
		Disease	1.961	0.018	4.257	0.201	0.997	<0.0001	71	0.012

(i) The PLE-I model fitted to the MFGC abundances extremely well (significant with *p*-value < 0.0001), and this indicates the ubiquitous applicability of the PLE for assessing the metagenome spatial (i.e., inter-subject) heterogeneity of either MFGCs (this section) or MG (previous section).
(ii) MFGC-I and MFGC-II exhibited slightly different scaling parameter (*b*) values. The scaling parameter *b* of PLE-I ranged [2.027, 2.138] for MFGC-I and [1.715, 1.988] for MFGC-II, indicating that the MFGC-I has a higher heterogeneity degree. This difference should be due to their definitional difference: MFGC-I ignored the information of individual gene abundances, only taking into account the number of gene species (kinds), or gene *richness*. Obviously, ignoring the gene abundance information should lead to larger heterogeneity (difference), which explains why the PLE-I parameter *b* of MFGC-I was slightly higher (*b* > 2), while that of MFGC-II was lower (*b* < 2). Furthermore, MFGC-II should better embody functional redundancy information given that it considers both gene species (kinds or richness) and abundances.
(iii) Although MFGC-I and MFGC-II displayed slightly different ranges in their parameter *b*, the *b*-values from two databases (eggNOG and KEGG) within each MFGC type were rather close with each other and the difference was negligible. This simply indicates that the heterogeneity scaling based on metabolic pathways (KEGG database) or protein functions (eggNOG) makes little difference. This should be expected given that, at the MFGC level, both eggNOG and KEGG should be controlled by the same underlying gene-level mechanisms.
(iv) As shown in Table 5, in terms of the parameter changes associated with diseases, only IBD treatment displayed significant difference from its healthy control in MFGC-I, and the other diseases treatments did not exhibit any significant differences from their healthy controls. This result suggests that at the MFGC level, the metagenome spatial heterogeneity is less sensitive to diseases than at the MG level, as indicated by the randomization test results in Table 2.

**TABLE 5:** The *p*-value of the randomization test for the difference between the healthy and diseased treatments in their PLE-I (type-I power law extension) parameters in terms of the MFGC.

MFGC Type and Databases used	Microbiome	Treatments	*b*	ln(*a*)	*m*_0_
MFGC Type-I (eggNOG)	Obesity	Lean vs. Overweight	0.347	0.345	0.348
	Type 2 diabetes	Healthy vs. Disease	0.985	0.937	0.965
	IBD	Healthy vs. Disease	0.039	0.033	0.059
MFGC Type-I (KEGG)	Obesity	Lean vs. Overweight	0.442	0.465	0.444
	Type 2 diabetes	Healthy vs. Disease	0.987	0.913	0.947
	IBD	Healthy vs. Disease	0.018	0.012	0.025
MFGC Type-II (eggNOG)	Obesity	Lean vs. Overweight	0.421	0.388	0.432
	Type 2 diabetes	Healthy vs. Disease	0.551	0.556	0.597
	IBD	Healthy vs. Disease	0.330	0.370	0.375
MFGC Type-II (KEGG)	Obesity	Lean vs. Overweight	0.361	0.337	0.382
	Type 2 diabetes	Healthy vs. Disease	0.781	0.771	0.787
	IBD	Healthy vs. Disease	0.370	0.427	0.418

### MFGC-Level (Inter-MFGC) Spatial Heterogeneity in Terms of the MFGC (Metagenome Functional Gene Cluster) Distribution Measured With PLE-III

In the previous section, we investigated the spatial heterogeneity of MFGC by using the PLE-I (type-I power law extension) modeling. That is to analyze the inter-subject heterogeneity of their metagenomes in terms of the functional gene cluster (i.e., MFGC). In this section, we investigate the spatial heterogeneity at the MFGC-level by using PLE-III (type-III power law extension). In other words, by assuming that there exists an average MFGC in a mixed-MFGC setting (by ignoring the difference among MFGCs), we assess the heterogeneity of MFGCs at the average MFGC level. Therefore, a fundamental difference between the analysis here and the analysis in the previous section is that here, the heterogeneity is measured in terms of a virtually averaged MFGC (or at MFGC-level), while in the previous section, the heterogeneity was measured in terms of the whole metagenome (or at metagenome level).

To save page space, the results for MFGC-level spatial heterogeneity were listed in Supplementary Table S2 in the OSI (online Supplementary Information), from which we summarize the following findings:

(i) The PLE-III model fitted to the MFGC tables extremely significant with *p*-value < 0.0001 in all three case studies, and this indicates the ubiquitous applicability of the PLE for assessing the spatial (i.e., inter-subject) heterogeneity of an ‘averaged’ MFGC.
(ii) The scaling parameter (*b*) of the PLE-III model ranged narrowly [1.472, 1.654], and varied little either between the MFGC-I and MFGC-II or between the healthy and diseased treatments within each case study. This suggests that, the sensitivity of the scaling parameter (*b*) of PLE-III to host factors such as diseases is rather muted, and consequently may be of limited practical applications.
(iii) Contrary with the pattern of PLE-I in the previous section, where MFGC-I has slightly larger scaling parameter (*b*) value than MFGC-II has, here MFGC-I [1,472, 1.547] has slightly smaller *b*-value than MFGC-II [1.525, 1.654] does.
(iv) The scaling parameter (*b*) of PLE-III, estimated with KEGG or eggNOG showed little differences, similar with the previous PLE-I model.
(v) We also performed the randomization tests for the PLE-III parameters (Supplementary Table S3). In most cases, the model parameters did not showed significant differences between the healthy and diseased treatments.

## Conclusion and Discussion

In previous sections, we demonstrated that PLE-I and PLE-III, originally designed to measure community spatial heterogeneity and mixed-species population spatial aggregation in community ecology (Taylor, 1961, 1984; Ma, 2015), can be introduced to (*i*) measure *metagenome spatial heterogeneity* in terms of either MG or MFGC abundance distribution and measured with PLE-I; or (*ii*) MG-level (or MFGC-level) spatial heterogeneity measured with PL-III. The first application is a measure at the whole metagenome (more accurately, metagenome assemblage) level, because it measures the inter-subject (spatial) heterogeneity within a cohort or population of individuals in their metagenomes. The second application is a measure at MG or MFGC level, because it measures the inter-MG or inter-MFGC heterogeneity from the perspective of a virtually averaged MG or MFGC. Although we used the metagenomic datasets of the human microbiome to demonstrate the concepts and modeling analyses, the approaches should be equally applicable to the metagenomes of other microbiomes on the planet.

Traditionally, studies on heterogeneity have been mostly focused on population level, and metrics for community heterogeneity are relatively fewer. This may be to do with that community level studies are mostly focused on community diversity, instead. Nevertheless, heterogeneity and diversity are not the same (Shavit et al., 2016). First, the heterogeneity is a “group” property, while diversity can be measured with one individual or single community. Without comparing two entities, heterogeneity does not make sense. Second, diversity is a measure of numbers (and relative abundances), while heterogeneity needs to be measured by interactions and working together (Shavit et al., 2016). For example, one may say, “zoos are diverse, and natural ecosystems are heterogeneous” (Ayelet Shavit and Aaron Ellison, personal communication, 22 April 2020).

Two additional heterogeneity indexes that can be used to measure community level heterogeneity are: (*i*) Variance/mean ratio (*V/m*) and (*ii*) community dominance index *M^∗^/m*. Both are counterparts of spatial aggregation index and patchiness index at the population level in population ecology (Taylor, 1984; Ma, 1991). Both indexes have different definitions and interpretations. The *V/m* heterogeneity index is simply a ratio of the mean species abundances (*m*) and corresponding variance (*V*), and a larger index value indicates higher heterogeneity (Taylor, 1984; Ma, 1991). The community dominance index (*D*_*c*_) was defined by Ma and Ellison (2018), and a larger index value indicates lower heterogeneity. Table 6 shows the values of the computed heterogeneity indexes for the three datasets (see Supplementary Table S1) we used, as well as the *p*-values from Wilcoxon tests for the differences between the healthy and diseased treatments in each of the three datasets.

**TABLE 6:** The *p*-value of Wilcoxon tests for the difference between the healthy and diseased treatments in their metagenome spatial heterogeneities and community dominance (also see Supplementary Figure S1A for the V/M heterogeneity index and Supplementary Figure S1B for the community dominance index).

Taylor’s Power Law Extension (TPLE)	Case Study	Treatments	Mean of Healthy	Mean of Diseased	*P-value*
Variance/mean-ratio heterogeneity Index (*V*/*m*)	Obesity	Lean vs. Overweight	886.50	1129.0	<0.001
	Type-2 Diabetes	Healthy vs. Disease	594.10	761.10	<0.001
	IBD	Healthy vs. Disease	786.70	1064.4	<0.001
Community dominance Index (*M^∗^/m*)	Obesity	Lean vs. Overweight	45.717	39.910	<0.001
	Type-2 Diabetes	Healthy vs. Disease	27.766	34.444	<0.001
	IBD	Healthy vs. Disease	28.419	37.765	<0.001

Obviously, the two heterogeneity indexes described above are much simpler to implement than the power law modeling introduced in this study. Furthermore, both indexes displayed significant differences between the healthy and diseased treatments in their metagenome heterogeneity. Given their simplicity, a natural question is: what are the advantages from using the power law modeling? The answer is that the power law analysis we presented offers tools to synthesize and measure the heterogeneity at various scales (MG, MFGC) across individuals in a cohort (population), with a unified power law model, which achieved the rare status of classic ecological laws. In fact, the power law analysis demonstrated here can also be applied to measure metagenome temporal stability at similar scales to the previous spatial versions. Furthermore, the power law analysis provides a unified modeling tool to assess and interpret the heterogeneity from ecological, taxonomical, functional and evolutionary perspectives, because it can be applied to both OTU tables and MGA tables, with the exactly same mathematical model (the power law model). When using MGA tables, it can be universally applied to the scales of the metagenomic gene or metagenome functional gene cluster.

Perhaps an even more compelling case for using the TPL/PLE parameters rather than the simple heterogeneity indexes has to do with the difference between heterogeneity and diversity. As argued previously, heterogeneity is a “group” property; measuring heterogeneity requires at least two entities. While TPL/PLE can synthesize the information from potentially unlimited number of entities (samples), the two heterogeneity indexes previously introduced were computed from single sample. To synthesize information from multiple samples, additional statistics such as the *mean* of the heterogeneity values, as displayed in Table 6, must be used. Nevertheless, the distribution of heterogeneity values may satisfy the Gaussian distribution. This may make the usage of mean problematic since the distribution of heterogeneity *per se* is usually highly skewed and follows power law distribution. In addition, it may even argue that the two simple heterogeneity indexes are similar to diversity measures. For example, the community dominance (*M^∗^/m*), as heterogeneity index, may even be treated as the other side of the evenness (diversity) coin (Ma and Ellison, 2018).

## Data Availability Statement

The datasets analyzed in this manuscript are publically available for downloading and the sources were noted in Supplementary Table S1.

## Author Contributions

ZM designed the study, conducted the study and wrote the manuscript.

## Conflict of Interest

The author declares that the research was conducted in the absence of any commercial or financial relationships that could be construed as a potential conflict of interest.

## Footnotes

1 http://hannonlab.cshl.edu/fastx_toolkit/
