# Supplementary material for: Assessing and Interpreting the Metagenome Heterogeneity With Power Law
Source: Front Microbiol. 2020 May 6;11:648. doi: 10.3389/fmicb.2020.00648 (PMC7218080; doi:10.3389/fmicb.2020.00648)
Supplement: Supplementary file 1 [file Data_Sheet_1.pdf]

**Online Supplementary Information (OSI) for** Ma ZS (2020) Assessing and interpreting the heterogeneity of human gut metagenome with power law. *Frontiers in Microbiology*

**Including**: Table S1-S3; Fig S1; Help-file for R-Scripts; R-Scripts for Power Law Analysis and Randomization Tests.

**Table S1.** Brief information on the 3 case studies (datasets) used to demonstrate the PLEs

| **Cases** | **Groups** | **Number of Samples** | **Number of Samples Used*** | **References** |
|---|---|---|---|---|
| Obesity | Lean | 96 | 96 | Qin *et al*. 2010<br>Chatelier *et al*. 2013 |
| | Obesity | 17 | 0 (not used) | |
| | Overweight | 168 | Randomly sampled 96 | |
| Type-II Diabetes | Healthy | 74 | 74 | Qin *et al*. 2012 |
| | Diseased | 71 | 71 | |
| IBD | Healthy | 24 | 71 | Nielsen *et al*. 2014 |
| | Healthy Relative | 47 | | |
| | CD | 21 | 0 (not used) | |
| | UC | 127 | Randomly sampled 71 | |

*To keep balanced sample sizes between the healthy and diseased treatments, we discarded the "lean" and "CD" groups because of their small sample sizes. For "overweight" and "UC" groups, we randomly chose 96 and 71 samples respectively to keep the balanced (the same or rather close) sample sizes.

**Table S2**. The parameters of PLE-III (type-III power law extension) models for MFGC spatial heterogeneity, fitted with the MFGC (metagenome functional gene cluster) tables

| **MFGC Type & Database Used** | **Microbiome** | **Treatments** | ***b*** | **SE(*b*)** | **ln(*a*)** | **SE[ln(*a*)]** | ***R*** | ***p*-value** | ***N*** | ***CACD*** |
|---|---|---|---|---|---|---|---|---|---|---|
| | | | | | | | | | | |
| **MFGC Type-I (eggNOG)** | Obesity | Lean | 1.519 | 0.014 | 0.309 | 0.059 | 0.985 | 0.0000 | 354 | 0.551 |
| | | Overweight | 1.485 | 0.015 | 0.372 | 0.061 | 0.983 | 0.0000 | 354 | 0.464 |
| | Type 2 diabetes | Healthy | 1.478 | 0.016 | 0.425 | 0.066 | 0.981 | 0.0000 | 327 | 0.411 |
| | | Disease | 1.547 | 0.016 | 0.501 | 0.064 | 0.983 | 0.0000 | 320 | 0.400 |
| | IBD | Healthy | 1.523 | 0.015 | 0.246 | 0.062 | 0.983 | 0.0000 | 348 | 0.625 |
| | | Disease | 1.472 | 0.014 | 0.413 | 0.058 | 0.984 | 0.0000 | 350 | 0.417 |
| | | | | | | | | | | |
| **MFGC Type-I (KEGG)** | Obesity | Lean | 1.507 | 0.020 | 0.271 | 0.069 | 0.977 | 0.0000 | 287 | 0.586 |
| | | Overweight | 1.491 | 0.020 | 0.269 | 0.072 | 0.975 | 0.0000 | 283 | 0.578 |
| | Type 2 diabetes | Healthy | 1.502 | 0.020 | 0.421 | 0.070 | 0.978 | 0.0000 | 255 | 0.433 |
| | | Disease | 1.545 | 0.019 | 0.596 | 0.067 | 0.981 | 0.0000 | 262 | 0.335 |
| | IBD | Healthy | 1.506 | 0.021 | 0.235 | 0.073 | 0.975 | 0.0000 | 280 | 0.629 |
| | | Disease | 1.495 | 0.020 | 0.246 | 0.069 | 0.977 | 0.0000 | 276 | 0.608 |
| | | | | | | | | | | |
| **MFGC Type-II (eggNOG)** | Obesity | Lean | 1.634 | 0.012 | 2.981 | 0.078 | 0.991 | 0.0000 | 354 | 0.009 |
| | | Overweight | 1.589 | 0.010 | 3.033 | 0.067 | 0.993 | 0.0000 | 354 | 0.006 |
| | Type 2 diabetes | Healthy | 1.558 | 0.010 | 2.674 | 0.061 | 0.994 | 0.0000 | 327 | 0.008 |
| | | Disease | 1.532 | 0.010 | 2.899 | 0.065 | 0.993 | 0.0000 | 320 | 0.004 |
| | IBD | Healthy | 1.635 | 0.010 | 2.532 | 0.065 | 0.994 | 0.0000 | 348 | 0.019 |
| | | Disease | 1.595 | 0.009 | 2.778 | 0.058 | 0.994 | 0.0000 | 350 | 0.009 |
| | | | | | | | | | | |
| **MFGC Type-II (KEGG)** | Obesity | Lean | 1.650 | 0.013 | 2.830 | 0.069 | 0.991 | 0.0000 | 287 | 0.013 |
| | | Overweight | 1.625 | 0.013 | 2.867 | 0.070 | 0.991 | 0.0000 | 283 | 0.010 |
| | Type 2 diabetes | Healthy | 1.605 | 0.013 | 2.617 | 0.067 | 0.991 | 0.0000 | 255 | 0.013 |
| | | Disease | 1.586 | 0.013 | 2.704 | 0.065 | 0.991 | 0.0000 | 262 | 0.010 |
| | IBD | Healthy | 1.654 | 0.013 | 2.509 | 0.067 | 0.992 | 0.0000 | 280 | 0.022 |
| | | Disease | 1.630 | 0.013 | 2.690 | 0.066 | 0.992 | 0.0000 | 276 | 0.014 |

**Table S3**. The *p*-value of the randomization test for the difference between the healthy and diseased treatments in their PLE model parameters fitted with the MFGC tables

| **Power Law Extension (PLE)** | **Microbiome** | **Treatments** | ***b*** | **ln(*a*)** | ***CACD*** |
|---|---|---|---|---|---|
| **MFGC Type-I (eggNOG)** | | | | | |
| Type-I PLE for Metagenome Spatial Heterogeneity | Obesity | Lean vs. Overweight | 0.347 | 0.345 | 0.348 |
| | Type 2 diabetes | Healthy vs. Disease | 0.985 | 0.937 | 0.965 |
| | IBD | Healthy vs. Disease | 0.039 | 0.033 | 0.059 |
| Type-III PLE for MFGC Spatial Aggregation | Obesity | Lean vs. Overweight | 0.122 | 0.31 | 0.163 |
| | Type 2 diabetes | Healthy vs. Disease | 0.006 | 0.296 | 0.827 |
| | IBD | Healthy vs. Disease | 0.059 | 0.012 | 0.001 |
| **MFGC Type-I (KEGG)** | | | | | |
| Type-I PLE for Metagenome Spatial Heterogeneity | Obesity | Lean vs. Overweight | 0.442 | 0.465 | 0.444 |
| | Type 2 diabetes | Healthy vs. Disease | 0.987 | 0.913 | 0.947 |
| | IBD | Healthy vs. Disease | 0.018 | 0.012 | 0.025 |
| Type-III PLE for MFGC Spatial Heterogeneity | Obesity | Lean vs. Overweight | 0.434 | 0.958 | 0.911 |
| | Type 2 diabetes | Healthy vs. Disease | 0.039 | 0.028 | 0.071 |
| | IBD | Healthy vs. Disease | 0.636 | 0.869 | 0.792 |
| **MFGC Type-II (eggNOG)** | | | | | |
| Type-I PLE for Metagenome Spatial Heterogeneity | Obesity | Lean vs. Overweight | 0.421 | 0.388 | 0.432 |
| | Type 2 diabetes | Healthy vs. Disease | 0.551 | 0.556 | 0.597 |
| | IBD | Healthy vs. Disease | 0.33 | 0.37 | 0.375 |
| Type-III PLE for MFGC Spatial Heterogeneity | Obesity | Lean vs. Overweight | 0.007 | 0.901 | 0.175 |
| | Type 2 diabetes | Healthy vs. Disease | 0.153 | 0.05 | 0.015 |
| | IBD | Healthy vs. Disease | 0.182 | 0.013 | 0.025 |
| **MFGC Type-II (KEGG)** | | | | | |
| Type-I PLE for Metagenome Spatial Heterogeneity | Obesity | Lean vs. Overweight | 0.361 | 0.337 | 0.382 |
| | Type 2 diabetes | Healthy vs. Disease | 0.781 | 0.771 | 0.787 |
| | IBD | Healthy vs. Disease | 0.37 | 0.427 | 0.418 |
| Type-III PLE for MFGC Spatial Heterogeneity | Obesity | Lean vs. Overweight | 0.107 | 0.721 | 0.341 |
| | Type 2 diabetes | Healthy vs. Disease | 0.242 | 0.378 | 0.146 |
| | IBD | Healthy vs. Disease | 0.33 | 0.02 | 0.048 |

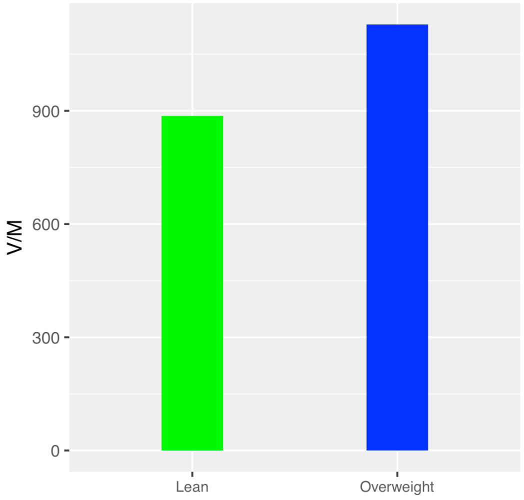


(**Fig** S1A)

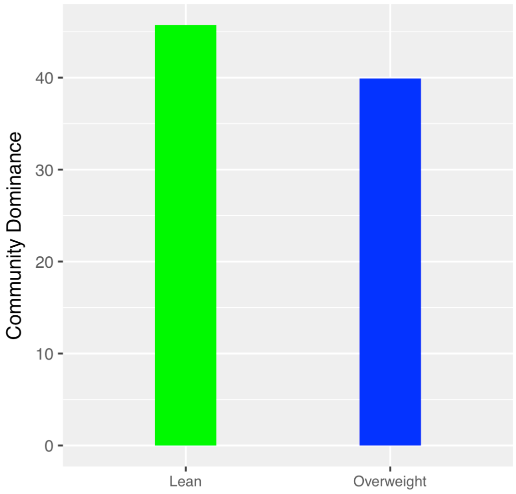


(**Fig** S1B)

**Fig S1**. The charts showing the differences between the lean and overweight groups by two heterogeneity indexes: the left is V/M heterogeneity index (Fig S1A) and the right is the community dominance index (Fig S1B)

# Help file for "PowerLaw.r" and "PowerLaw-Randomization.r" R-Scripts

## PowerLaw.r: Computing the parameters of the power law extensions (PLEs)

(1) Program Installation: The R version used is Version R3.5.1. The R-Script (PowerLaw.r) and its input data (*e.g.*, "DemoData.txt") should be located in the same folder.

(2) Input File
The input file is an MGA (metagenomic gene abundance) table, an MxN matrix with elements representing the MG abundance (copy numbers for each MG), separated by "\t" (Tab) symbol, where M is the number of metagenome samples, and N is the number of MGs. The first row lists the gene names (numbering), and the first column lists sample IDs or names.

(3) Usage
Copy the R-script (PowerLaw.r) and input file (e.g., "DemoData.txt") to the same folder. Enter the following command under Linux shell prompt:

```
$ Rscript PowerLaw.r DemoData.txt n
```

where "DemoData.txt" is the MGA table. "n" specifies the PLE, 1 for Type-I PLE, 2 for Type-2 PLE, 3 for Type-3 PLE, and 4 for Type-4 PLE.

(4) Output File (*e.g.* "Type-1-PLE.txt")
The output from the R-script "PowerLaw.r" is stored in "Type-1-PLE.txt". It contains: *b*, ln(*a*), CACD, *R*, *p*-value, *N*. The first three are the PLE parameters, and the last three are parameters indicating the goodness-of-fitting to the PLE model.

## PowerLaw-Randomization.r: Performing the randomization tests for Type-I PLE

(1) Program Installation: The R version used in this code is version R3.5.1. The R-script (PowerLaw-Randomization.r) and its input data (*e.g.*, "DemoData1.txt" and "DemoData2.txt") should be located in the same folder.

(2) Input File
There are two input files, but both have the same format as the input file for the previous "PowerLaw.r" program.

(3) Usage
Copy the R-script (PowerLaw-Randomization.r) and input files to the same folder. Enter the following command under Linux shell prompt:

```
$ Rscript PowerLaw-Randomization.r DemoData1.txt DemoData2.txt
```

where "DemoData1.txt" and "DemoData2.txt" are the MGA tables.

(4) Output File ("Randomization-p-value.txt")
The output file contains the randomization test result for *b*, ln(*a*), CACD. There are five columns in this file. The first column is the parameter names. The second is the *D*-values of parameters between DemoData1 and DemoData2. The third and fourth columns are lower and upper limits of 95% confidence. The last column is the *p*-value of the randomization test. If $p>0.05$, the parameter has no significant difference between DemoData1 and DemoData2. If $p\leq0.05$, the parameter has significant difference between DemoData1 and DemoData2.

```
#Fitting TPL/PLE (Taylor's power law)/(Power law extensions)
#Useage: Rscript Powerlaw.r filename n

Power.law<-function(data,Type){
	#get the name of samples
	ID<-as.vector(data[,1])
	da<-data[,2:ncol(data)]
	cs<-apply(da,2,sum)
	da<-da[cs>0]
	#type I
	if((Type==1)|(Type==2)){
		Means=apply(da,1,function(x){mean(x)})
		Vars=apply(da,1,function(x){sum((x-mean(x))^2)/length(x)})
		Res=cbind(Means,Vars)
	}
	#type III
	if((Type==3)|(Type==4)){
		Means=apply(da,2,function(x){mean(x)})
		Vars=apply(da,2,function(x){sum((x-mean(x))^2)/length(x)})
		Res=cbind(Means,Vars)
	}

	Res=Res[Res[,2]!=0,]
	Res=Res[Res[,1]!=0,]
	x<-log(Res[,1])
	y<-log(Res[,2])

	fit<-lm(y~x)
	sf<-summary(fit)
	b<-sf$coefficients[2]
	SE.b<-sf$coefficients[4]
	lna<-sf$coefficients[1]
	SE.a<-sf$coefficients[3]
	r<-sqrt(sf$r.squared)
	fs<-sf$fstatistic
	p<-1-pf(fs[1],fs[2],fs[3])
	n<-(sf$df[2])+2
	cacd<-exp(lna/(1-b))
	res<-cbind(b,lna,cacd,r,p,n)

	return(res)
}

################################################################
args=commandArgs(T)
ID=args[1]
type=as.numeric(args[2])
otu=read.table(paste(ID,sep=""),header=T,sep="\t")
result<-Power.law(otu,type)
write.table(result,paste("Type-",type,"-PLE.txt",sep=""),quote=F,sep="\t",col.names=T,row.names=F)
```

```
#Randomization tests for the TPL/PLE parameters

Power.law<-function(Res){

	Res=Res[Res[,2]!=0,]
	Res=Res[Res[,1]!=0,]
	x<-log(Res[,1])
	y<-log(Res[,2])

	fit<-lm(y~x)
	sf<-summary(fit)
	b<-sf$coefficients[2]
	SE.b<-sf$coefficients[4]
	lna<-sf$coefficients[1]
	SE.a<-sf$coefficients[3]
	r<-sqrt(sf$r.squared)
	fs<-sf$fstatistic
	p<-1-pf(fs[1],fs[2],fs[3])
	n<-(sf$df[2])+2
	cacd<-exp(lna/(1-b))
	res<-cbind(b,lna,cacd,r,p,n)

	return(res)
}


##################################################################

#Main program
args=commandArgs(T)
input<-"data"
out<-"Randomization"
dir.create(out)
dam<-read.table(paste(args[1],sep=""),header=T,sep="\t",stringsAsFactors=F)
dan<-read.table(paste(args[2],sep=""),header=T,sep="\t",stringsAsFactors=F)

dam=dam[,2:ncol(dam)]
dan=dan[,2:ncol(dan)]
csm=apply(dam,2,sum)
dam=dam[csm>0]
csn=apply(dan,2,sum)
dan=dan[csn>0]

Meansm=apply(dam,1,function(x){mean(x)})
Varsm=apply(dam,1,function(x){sum((x-mean(x))^2)/length(x)})
Resm=cbind(Meansm,Varsm)

Meansn=apply(dan,1,function(x){mean(x)})
Varsn=apply(dan,1,function(x){sum((x-mean(x))^2)/length(x)})
Resn=cbind(Meansn,Varsn)

param=Power.law(Resm)
paran=Power.law(Resn)
del=param-paran
del=c("raw",del)

param=c("raw",param)
paran=c("raw",paran)

#combined data
Res=rbind(Resm,Resn)
for(i in 1:2){
		print(i)
		s<-sample(1:nrow(Res),nrow(Res))
```

```
        das<-Res[s,]
        pdam<-das[1:nrow(Resm),]
        pdan<-das[(nrow(Resm)+1):nrow(Res),]

        #compute predicted delta type I
        plm<-Power.law(pdam)
        pln<-Power.law(pdan)
        pdel<-plm-pln
        pdel=c(i,pdel)
        del=rbind(del,pdel)

        param=rbind(param,c(i,plm))
        paran=rbind(paran,c(i,pln))

}
cn=c("index","b","lna","cacd","r","p","n")
write.table(del,paste(out,"/","delta-value.txt",sep=""),quote=F,sep="\t",col.names=cn,row.names=F)
del=read.table(paste(out,"/","delta-value.txt",sep=""),header=T,sep="\t")
da=del[,2:4]
p<-apply(da,2,function(x){a=x[1];b=x[2:length(x)];aa=abs(a);ab=abs(b);p=length(ab[ab>=aa])/length(b);lower=mean(b)-
sd(b)*1.96;upper=mean(b)+sd(b)*1.96;res=cbind(a,lower,upper,p)})
p=t(p)
p=cbind(colnames(del)[2:4],p)

#write.table(p,paste(out,"/","p-
value.txt",sep=""),quote=F,sep="\t",col.names=c("name","del","lower","upper","p"),row.names=F)
#write.table(param,paste(out,"/","random-",args[1],sep=""),quote=F,sep="\t",col.names=cn,row.names=F)
#write.table(paran,paste(out,"/","random-",args[2],sep=""),quote=F,sep="\t",col.names=cn,row.names=F)
system('rm -rf Randomization')
write.table(p,"Randomization-p-value.txt",quote=F,sep="\t",col.names=c("name","del","lower","upper","p"),row.names=F)
```
